# Tumor-Related Trigeminal Neuralgia: Clinical Features, Pathophysiology, and the Contemporary Role of Radiosurgery in Tumor Control and Pain Relief

**DOI:** 10.7759/cureus.116049

**Published:** 2026-09-10

**Authors:** Jesus Antonio Rodriguez Santos, Jennifer De Alas, Eduardo E Lovo

**Affiliations:** 1 Department of Radiosurgery, Centro Internacional de Cancer, San Salvador, SLV; 2 Neurosurgery-Gamma Knife Program, Centro Internacional de Cancer, San Salvador, SLV

**Keywords:** gamma knife, microsurgery, petroclival meningioma, secondary trigeminal neuralgia, skull base, stereotactic radiosurgery, trigeminal neuralgia

## Abstract

Trigeminal neuralgia arising from an intracranial tumor is uncommon but therapeutically distinctive because the responsible lesion is simultaneously the generator of facial pain and an independent oncological problem. When anatomically feasible, microsurgical treatment remains the preferred approach for achieving rapid, deep, and durable pain relief, particularly when decompression of the trigeminal nerve can be achieved. However, a clinically important subset of patients may be unsuitable for open surgery because of age, fitness, comorbidity, tumor anatomy, or operative risk, while others may decline surgical intervention. For these patients, radiosurgery alone represents an important noninvasive treatment strategy, although its role in pain control differs from its well-established role in tumor control. This narrative review synthesizes a purposively assembled core evidence base of 14 studies comprising comparative cohorts reporting both microsurgical and radiosurgical arms, contemporary radiosurgical series reporting pain outcomes by target, and systematic reviews and meta-analyses synthesizing either modality. The review examines tumor control and pain relief as separate therapeutic endpoints and defines what the available literature does and does not permit to be concluded. Pain outcomes are reported using each source’s own threshold and are not converted between scales, and overlapping cohorts are identified and never aggregated. Across comparative cohorts and secondary pooled analyses, microsurgery was associated with a higher proportion of favorable pain outcomes and, in one meta-analysis, a longer median time to pain recurrence, with equivalent tumor control, principally in petroclival and petrous apex meningioma. Radiosurgery achieves excellent tumor control but more modest, delayed, and attenuating pain relief. Its principal role is therefore not to replace microsurgical treatment in otherwise suitable surgical candidates but to provide a noninvasive option when surgery is unsuitable, carries substantial anatomical or medical risk, or is declined. Radiosurgical pain relief cannot be explained solely by tumor volume reduction, and its mechanism remains unresolved. The characteristic radiosurgical complication is trigeminal sensory dysfunction, which is frequently permanent. Outcome definitions varied between studies, the cohorts were not independent, and none of the comparisons was randomized; therefore, observed associations cannot be interpreted as causal and may be influenced by confounding by indication. The optimal radiosurgical target remains unresolved, with every combined-target series comprising fewer than 20 patients. Anatomy-driven individualized selection, incorporating tumor-nerve relationships, surgical candidacy, nerve visibility, and the feasibility of alternative or staged treatment, remains the most defensible current approach.

## Introduction and background

Trigeminal neuralgia is a disorder of sudden, severe, electric shock-like pain in the face, arising in the territory of the trigeminal nerve, the nerve that carries sensation from the face to the brainstem. In most patients, no tumor is found, and the pain is attributed either to a blood vessel compressing the nerve where it leaves the brainstem, termed “classical trigeminal neuralgia,” or to no identifiable cause, termed “idiopathic trigeminal neuralgia.” In a minority, an intracranial tumor is demonstrated, and the neuralgia is then classified as secondary. The International Classification of Headache Disorders divides trigeminal neuralgia into these three forms, the last requiring demonstration of an underlying causative condition [1,2]. The tumors responsible are usually benign and lie outside the substance of the brain, that is, extra-axial: in the space between the cerebellum and the pons through which the cranial nerves emerge, known as the cerebellopontine angle; along the junction between the petrous temporal bone and the clivus, the petroclival region and petrous apex; or within the two dural pockets traversed by the trigeminal nerve and the internal carotid artery, Meckel’s cave and the cavernous sinus [2,3].

One distinction governs the interpretation of this entire literature and is therefore made at the outset. Tumor-related trigeminal neuralgia denotes brief, paroxysmal, stabbing unilateral facial pain satisfying the diagnostic criteria for trigeminal neuralgia in a patient with a demonstrated causative lesion. Tumor-related trigeminal neuropathy denotes sensory dysfunction of the same nerve, that is, numbness, altered sensation, or continuous aching discomfort, without such paroxysmal pain. The two behave differently after nerve-directed treatment, and their conflation in primary reports is a recognized source of distortion: one systematic review excluded studies reporting sensory dysfunction without documented paroxysmal pain [2], and one meta-analysis excluded four otherwise eligible studies because the two entities could not be separated [3]. Throughout this review, the term “tumor-related trigeminal neuralgia” is reserved for the paroxysmal syndrome.

So defined, the condition sits between two well-developed literatures: it is neither classical trigeminal neuralgia, in which the therapeutic target is unambiguous, nor asymptomatic skull base tumor management, in which oncological control is the sole endpoint [1,2]. Neither body of work answers the question that arises at the bedside, which is what to treat when the lesion generating the pain is simultaneously a tumor requiring control in its own right.

The central problem addressed here is that tumor control and pain control behave as separable endpoints. In the studies discussed below, tumor-targeted radiosurgery has achieved growth control in nearly every treated patient, while complete initial pain relief was obtained in a much smaller fraction [4], and in the largest single-institution comparison available, tumor control was reported as identical in the two arms, while medication-free pain relief differed more than twofold [5]; the corresponding figures are given in the relevant sections below rather than here. Any framework treating radiographic stability as a proxy for symptomatic success will therefore mislead, and this distinction is assumed throughout.

This review was prompted not only by the heterogeneity of the published literature but also by the recurring difficulty of applying it. In a regional practice where tumor-related trigeminal neuralgia is uncommon and specialized radiosurgical care is concentrated in few centers, each case requires a decision about what to irradiate, in what sequence, and within how many treatment episodes, decisions for which the published evidence offers limited and, as shown below, partly contradictory guidance.

The objective of this review is to set out, for adults with trigeminal neuralgia attributable to an intracranial tumor, what the available literature supports regarding pain relief and tumor control after microsurgical resection and after stereotactic radiosurgery, and how the two should be weighed in an individual patient. Four questions are addressed: how the condition presents and which tumors are responsible; what is and is not known about how the pain is generated and how treatment relieves it; how microsurgery and radiosurgery compare on pain relief, durability, tumor control, and morbidity; and which anatomical and clinical features should govern selection between them, including the unresolved question of the radiosurgical target. The population considered is adults with a demonstrated causative tumor and paroxysmal facial pain; the interventions are microsurgical resection and stereotactic radiosurgery directed at the tumor, at the trigeminal nerve, or at both; the comparator, where one exists, is the alternative modality; and the outcomes are pain relief by each source’s own threshold, time to relief and to recurrence, tumor control, and treatment-related morbidity. The intended contribution is a critical and explicitly non-systematic appraisal that keeps tumor control and pain relief separate, declines to convert between incompatible outcome scales, and identifies overlapping cohorts so that the same patients are not counted twice.

## Review

Scope of this review and selection of the literature

This is an expert narrative review. The literature was assembled purposively by the authors rather than by a protocol-driven search: from the published work addressing trigeminal neuralgia of tumoral origin, we selected the comparative cohorts reporting both microsurgical and radiosurgical arms, the contemporary radiosurgical series reporting pain outcomes by target, and the published systematic reviews and meta-analyses of either modality, together with one large microsurgical cohort providing contextual operative safety data and one narrative source covering classification and medical therapy. We concentrated on work reporting a defined pain outcome in patients with a demonstrated causative tumor, except where a source is cited for context.

What this review does not do should be stated plainly. No search protocol was registered or applied; no databases, search terms, date limits, or language restrictions were specified in advance; screening was not performed in duplicate; and no study-selection flow diagram, list of excluded full-text articles, or formal risk-of-bias appraisal, such as ROBINS-I for the primary comparative cohorts or AMSTAR 2 for the secondary syntheses, was undertaken. No quantitative pooling was performed. PRISMA was neither followed nor is it applicable because this is not a systematic or a scoping review. Comprehensive identification of the relevant literature is therefore not claimed, and the selection reflects the authors’ judgment of which studies bear most directly on the questions set out above. Readers requiring a reproducible appraisal of the whole field should consult the published systematic reviews and meta-analyses that appear in Table 1, each of which reports its own search strategy and eligibility criteria.

**Table 1:** Characteristics of the principal studies discussed in this review. Patient numbers are not additive across rows: several syntheses incorporate primary studies listed separately, as indicated in the final column. Where a synthesis reports different denominators for different variables, the total included population is given first. BNI, Barrow Neurological Institute pain intensity score; CPA, cerebellopontine angle; GKRS, Gamma Knife radiosurgery; REZ, root entry zone; SR, systematic review; SRS, stereotactic radiosurgery; TN, trigeminal neuralgia; TRTN, tumor-related trigeminal neuralgia; TS, trigeminal schwannoma; VS, vestibular schwannoma

Study	Design and evidence type	Patients (n)	Population	Modality and target	Overlap with other studies discussed
Hassooni et al. (2026) [1]	Narrative review; secondary	-	Secondary TN, all causes	All modalities; trigeminal REZ	None
Nugroho et al. (2023) [2]	SR without meta-analysis; secondary	27 studies; 517 patients (538 treatment episodes)	CPA tumors; epidermoid 43.7%, meningioma 29.8%, schwannoma 24.6%	Surgery 320; tumor SRS 196; nerve SRS 22	None
Hajikarimloo et al. (2025) [3]	SR + meta-analysis; secondary	19 studies; 454 patients (histology available for 449)	Meningioma 67.7%, VS 18.3%, TS 8.2%	SRS; tumor/nerve/dual	Contains refs [4,10] and SRS arms of [5,8]
Kano et al. (2011) [4]	Retrospective cohort; primary	12	Petroclival meningioma	GKRS; tumor; 13 Gy (11-16), 50% isodose	In refs [3,6,7]
Park et al. (2020) [5]	Consecutive comparative cohort; primary	70 (35/35)	Newly diagnosed petroclival meningioma <3.5 cm	Surgery vs GKRS (tumor); 14 Gy (6-15), 50% isodose	Both arms in ref [6]; SRS arm in ref [3]
Hallak et al. (2024) [6]	SR + meta-analysis; secondary	8 studies; 138 (57/81)	Petroclival meningioma	Surgery vs SRS; tumor 74, tumor + nerve 7	Contains refs [4,5]
Peciu-Florianu et al. (2021) [7]	SR + meta-analysis; secondary	13 series; 103-228 patients depending on outcome	Meningioma, vestibular schwannoma	SRS; tumor vs tumor + nerve	Contains ref [4]; shares most studies with ref [3]
Hegazy et al. (2022) [8]	Historical comparative cohort; primary	47 (22/25)	Petrous apex meningioma <3 cm; all BNI V at baseline	Surgery vs GKRS (tumor); 14 Gy (6-15), 55% isodose	SRS arm in ref [3]; surgical arm probably overlaps ref [6]
Sinha et al. (2026) [9]	Retrospective, session-level; primary	12 patients (7 TRTN)/17 sessions	TN with coexisting intracranial tumor	GKRS; nerve/TREZ; mean 72.8 Gy (60-80)	None
Hall et al. (2022) [10]	Retrospective series; primary	21 patients (27 treatment courses)	Benign 62%, malignant 38%	CyberKnife; nerve 5, tumor 10, both 6	In ref [3]
Murayi et al. (2025) [11]	Retrospective cohort; primary	17	Meningioma 71%, VS 24%, TS 6%; cisternal contact 94.1%	GKRS; tumor; 12-13 Gy or 25 Gy/5 fx	None
Rodriguez Santos et al. (2026) [12]	Case report; primary	1	Cavernous sinus meningioma with complete nerve encasement	Tumor 14 Gy + bilateral medial thalamus, Dmax 125 Gy	None
Peciu-Florianu et al. (2021) [13]	SR + meta-analysis; secondary	18 studies; 564 patients; TN outcome reported in 126	Trigeminal schwannoma	SRS; tumor	None
Lee et al. (2025) [14]	Contextual surgical safety cohort; no TN-specific outcome	1000	CPA tumors; VS 89%	Microsurgery	None

Three properties of this literature required explicit handling. First, several of the syntheses discussed here incorporate primary studies that are also discussed individually: one petroclival series contributes patients to three reviews [3,4,6,7], both arms of one comparative cohort are incorporated within one meta-analysis, where they supply the majority of its microsurgical group [5,6], and two meta-analyses share most of their source studies [3,7]. Overlap was identified by comparing the study list of each synthesis against the primary reports; patient numbers are never aggregated across overlapping sources, no two overlapping sources are presented as independent corroboration, and the overlap identified for each source is recorded in the final column of Table 1. Second, where a source reports the same quantity inconsistently in different parts of the same paper, or where denominators differ between variables within a single analysis, the discrepancy is reported rather than silently resolved, and the denominator applying to each figure is stated. Third, pain outcomes are reported using each source’s own threshold and are never converted between scales, because the Barrow Neurological Institute (BNI) pain intensity score [15] and the Visual Analog Scale (VAS) measure related but nonidentical quantities, and the thresholds in use across these studies are not interconvertible.

All quantitative values reproduced in this review are those published by the cited source. The authors performed no calculation, recalculation, pooling, subgroup analysis, or statistical comparison of any kind, and no estimate appearing here originates with us. Pooled proportions and CIs are those of the published meta-analyses, and the number of contributing studies and patients for each is given in Table 1; the model, heterogeneity statistic, and assessment of small-study effects are those of the source and are not reproduced here in full. Reported p-values are likewise those of the original studies, and, because they arise from nonrandomized comparisons and, in some instances, from comparisons made across separate cohorts, they should be read descriptively rather than as evidence from a valid comparative trial. Median times to relief and to recurrence are those reported by each source’s own survival analysis; censoring patterns and follow-up distributions are not described in these studies in a form permitting independent appraisal, so these medians should be read as summaries of the published analyses rather than as directly comparable quantities. The characteristics of the studies discussed in this review are summarized in Table 1, and their reported outcomes are presented in Table 2.

**Table 2:** Reported clinical and radiological outcomes of the studies discussed in this review. Outcome definitions differed substantially across studies and were not harmonized; consequently, the reported proportions should not be interpreted as directly comparable effect estimates. Pain outcome thresholds are stated exactly as defined by each source and are not converted between scales. ARE, adverse radiation effect; BNI, Barrow Neurological Institute pain intensity score; HB, House-Brackmann; TN, trigeminal neuralgia

Study	Principal outcome as defined by the study
Hassooni et al. (2026) [1]	Classification, diagnostic pathway, medical therapy
Nugroho et al. (2023) [2]	Improved 97.2% (surgery) vs 79.1% (tumor SRS); worse 0% vs 6.6%
Hajikarimloo et al. (2025) [3]	BNI I 38% (95% CI 27-50); BNI I-III 73% (63-83); ARE 14% (7-22)
Kano et al. (2011) [4]	BNI I 25% initial, 41.7% final; tumor control 100%
Park et al. (2020) [5]	BNI I-II 91% vs 37% (p < 0.01); tumor control 94% both
Hallak et al. (2024) [6]	Relief (BNI I-II) 82% (50-100) vs 31% (18-45), p = 0.004
Peciu-Florianu et al. (2021) [7]	BNI I-IIIb 83.8% initial, 41.2% maintained; recurrence 34.7%
Hegazy et al. (2022) [8]	Good BNI (I/II/IIIa) 86.4% vs 28% (p < 0.001)
Sinha et al. (2026) [9]	Improvement in 76.5% of sessions; numbness 25%, all mild
Hall et al. (2022) [10]	Median ΔBNI -1/0/-2 at last follow-up (p = 0.04 across targets)
Murayi et al. (2025) [11]	Pain free 35% at censored endpoint; median 4.6 months to improvement
Rodriguez Santos et al. (2026) [12]	VAS 0 by day 6, sustained 39 months
Peciu-Florianu et al. (2021) [13]	Tumor control 92.3%; TN improvement 63.5%; worsening 10.7%
Lee et al. (2025) [14]	CSF leak 10%; HB I/II 88-90%; no mortality

Three sources are used indirectly and are labeled as such wherever they appear. The contemporary series of 1000 cerebellopontine angle operations listed in Table 1 was not selected for trigeminal neuralgia and reports no pain outcome; it is cited solely for contextual operative-safety data and provides no direct evidence about this condition. The single published case report from the authors’ own institution is cited illustratively in the discussion of targeting where the trigeminal nerve cannot be identified; one patient cannot support a treatment recommendation. The narrative source listed first in Table 1 is cited for classification and for medical therapy rather than for treatment outcomes. Separately, the small institutional experience presented in a clearly demarcated section below is original observational data rather than part of the literature discussed here; it is reported at an explicitly lower evidential level, is never aggregated with any published series, and supports no comparative inference.

Clinical characteristics and tumor spectrum

Tumor-related trigeminal neuralgia is defined by paroxysmal unilateral facial pain fulfilling the criteria for trigeminal neuralgia in a patient with a demonstrated causative lesion [1,2]. The distinction from tumor-related trigeminal neuropathy, defined in the Introduction, has direct consequences for the interpretation of the literature: because neuropathic facial pain responds poorly to nerve-directed intervention, the conflation of the two entities in primary reports probably attenuates apparent treatment effects [2,3]. The inconsistency is visible in the primary literature itself: one comparative cohort in which every patient scored BNI V before treatment, that is, severe pain without relief, is reported under the heading of intractable trigeminal neuropathy while describing its population as having trigeminal neuralgia [8].

No single frequency figure can be defended, because three quantities are reported in similar language: tumors are identified in 1-13% of patients presenting with facial pain [2,9], trigeminal neuralgia develops in 2-12% of posterior fossa tumors [5] and approximately 5% of petroclival meningiomas [6], and approximately 15% of all trigeminal neuralgia is secondary to any cause [1]. Frequencies vary by an order of magnitude according to referral pattern.

Pain is predominantly maxillary and mandibular and frequently multidivisional, with combined V1/V2/V3 involvement being commonest at 39% in one consecutive cohort [5]. Patients reach neurosurgical attention severely affected and medically refractory, with the commonest pretreatment BNI score being IV [6]. Preexisting trigeminal sensory deficit is common, with hypesthesia in seven of 12 patients in one series [4] and facial numbness in 48% of another before radiosurgery [10], so the phenotype is not uniformly that of classical trigeminal neuralgia, and post-treatment sensory change cannot be confidently attributed to treatment where baseline status is undocumented.

Anatomy is most usefully framed by the site at which the tumor engages the nerve: where systematically recorded, contact was cisternal in 94.1%, within Meckel’s cave in 23.5%, and within the cavernous sinus in 11.8%, with categories being nonexclusive [11]. Among petroclival meningiomas, subclassification by dural attachment distinguishes upper clivus (49%), tentorial (37%), and petrous apex (14%) tumors, each with a characteristic direction of nerve displacement; upper clivus lesions are separated from the brainstem by a single arachnoid plane, promoting adhesion and neurovascular invasion [5]. The nerve is frequently not visualizable, unidentifiable in eight of 12 patients in one series [4] and completely encased in an extreme case [12], a finding that determines which treatments remain technically available.

Two large datasets disagree completely about which tumors are responsible. In a meta-analysis of 454 patients pooled from radiosurgical series, histology was available for 449, among whom meningioma accounted for 67.7%, vestibular schwannoma for 18.3%, and trigeminal schwannoma for 8.2%, with epidermoid cysts essentially absent; denominators in that analysis vary by variable, with histology being reported for 449 patients, sex for 402, and pain distribution for 188 [3]. Among 517 patients pooled predominantly from surgical series confined to the cerebellopontine angle, epidermoid cysts were commonest at 43.7%, followed by meningioma at 29.8% and schwannoma at 24.6%; in that synthesis, the treatment categories sum to 538 rather than 517 because patients treated with more than one modality are counted within each category, the 21-patient difference corresponding to 15 patients receiving tumor and nerve targeting in a single session and six receiving nerve-targeted radiosurgery after failed tumor-targeted treatment [2]. This reflects ascertainment rather than differing tumor biology: epidermoid cysts are managed almost exclusively by resection and rarely irradiated, whereas meningiomas and vestibular schwannomas are radiosurgically tractable. Neither distribution should be presented in isolation as the commonest cause. Trigeminal schwannoma is a distinct entity in which nerve-directed targeting is not conceptually available [13], and malignant disease is represented by eight patients in a single series [10].

Pathophysiology

Several observations are securely established and are summarized, alongside the merely proposed mechanisms, in Table 3. Tumoral encasement without compression is common, with 81 tumors having been found intraoperatively to encase the nerve with no sign of compression by tumor or artery, which demonstrates that mechanical compression is not a necessary condition for the syndrome; a vascular component was present in a substantial minority, with an offending vessel having been identified in 108 of 320 surgical patients; and residual adherent tissue produces failure, with two patients whose pain was unchanged having capsule undissected from the nerve and both improving immediately after re-exploration [2].

**Table 3:** Proposed pathophysiological mechanisms and the direct evidence available for each. Established observations are those directly recorded at surgery or on imaging in patients with tumor-related trigeminal neuralgia; proposed mechanisms are explanatory models for which no direct confirmation exists within the literature discussed here. The mechanism of pain relief after tumor-targeted radiosurgery is explicitly acknowledged as unknown [3,7,11].

Proposed mechanism	Available evidence	Direct evidence in tumor-related trigeminal neuralgia	Limitations
Direct mechanical compression of the trigeminal nerve	Tumor compressing the nerve is one of three reproducible intraoperative relationships; meningiomas tend to displace the nerve [2]	Yes - direct surgical observation [2]	Not a necessary condition: 81 tumors were found encasing the nerve with no sign of compression [2]
Tumoral encasement without compression	81 tumors encasing the nerve without compression by tumor or artery [2]	Yes - direct surgical observation [2]	Mechanism by which encasement generates pain is not established
Secondary vascular compression	Offending vessel identified in 108 of 320 surgical patients, 100 of whom underwent microvascular decompression [2]	Yes - direct surgical observation [2]	Outcome of the decompressed subgroup is not reported separately [2]
Adherent arachnoid and residual capsule	Two patients with capsule undissected from the nerve improved immediately after re-exploration [2]	Yes - direct surgical observation [2]	Very small numbers; extent of resection could not be analyzed against pain outcome [6]
Focal demyelination with ephaptic transmission	Invoked as the dominant explanatory model [1,2,6]	No	No histological, electrophysiological, or imaging confirmation in this condition; imported from classical trigeminal neuralgia
Chemical irritation by keratin content (epidermoid)	Proposed to explain pain in the absence of compression [2]	No	Not demonstrated biochemically; consistent with but not proven by the encasement observations
Radiation-induced demyelination of the nerve	Advanced as the mechanism of radiosurgical relief because volume reduction fails to account for it [3]	No	Post hoc hypothesis; if correct, argues for targeting the nerve rather than the tumor
Transient tumor expansion	Proposed to explain early post-radiosurgical pain exacerbation; pseudoprogression 2.2-37.5% in trigeminal schwannoma [13]	Indirect - radiological only	Pseudoprogression rates derive from a different histology and are not correlated with pain in the source data
Tumor volume reduction (decompression by shrinkage)	Rationale for tumor-targeted radiosurgery [4,5,8]	Evidence is against it	Shrinkage associated neither with relief nor with worsening [4]; tumors enlarged while patients improved [9]; volume reduction (18.6 months) far exceeded pain response (5.3 months) [7]

Most consequentially, tumor volume reduction does not appear to explain pain relief by itself: shrinkage was associated neither with initial relief nor with subsequent worsening in one series [4], three tumors enlarged radiographically while all three patients improved in another [11], and the interval to measurable volume reduction (18.6 months) considerably exceeded the interval to pain response (5.3 months) in a pooled analysis [7].

The principal explanatory models remain hypotheses and are set against the available direct evidence in Table 3. Compression is proposed to produce focal demyelination, rendering the nerve hyperexcitable and permitting ephaptic transmission [1,2,6], although no included study provides histological, electrophysiological, or imaging confirmation in this condition; the model is imported from classical trigeminal neuralgia. Radiation-induced demyelination of the nerve has been advanced as the mechanism of radiosurgical relief precisely because volume reduction fails to account for it [3].

The resulting tension is central to the targeting debate below: tumor-targeted radiosurgery is justified by decompression through volume reduction, yet relief neither requires nor tracks shrinkage, and the alternative explanation of incidental nerve irradiation, if correct, argues for directing treatment at the nerve. The mechanism of relief after tumor-targeted radiosurgery is explicitly acknowledged as unknown [3,7,11].

Diagnosis and initial management

Magnetic resonance imaging with a dedicated trigeminal protocol is required in any patient with trigeminal neuralgia, with features heightening suspicion of a secondary cause, including onset before fifty years, bilaterality, ophthalmic division involvement, objective sensory deficit, and continuous or dull pain [1]. Beyond diagnosis, imaging should address three questions with direct therapeutic consequence: high-resolution steady-state sequences define the tumor-nerve relationship and segment of contact [11]; trigeminal nerve visibility should be explicitly reported, since it determines whether nerve-directed radiosurgery is feasible [4,12]; and patency of Meckel’s cave should be documented, since its obliteration precludes transovale access and removes percutaneous techniques from the salvage repertoire [7,12].

Medical therapy with sodium channel-blocking anticonvulsants remains the initial treatment, with further agents including lamotrigine, gabapentinoids, and botulinum toxin type A [1]. Its efficacy specifically in tumor-related disease is unquantified within the literature discussed here, since every treated cohort considered here consists of patients in whom medical management had already failed [4,8,10,11], and the frequently cited failure rates derive from sources outside the studies reviewed. Medication independence deserves recognition as an endpoint rather than a background detail: freedom from medication was achieved in 88% of microsurgical patients vs 24% of radiosurgical patients in one cohort [5].

Microsurgical management

Reported pain relief after resection is high across every series, although thresholds differ and are not interchangeable: any improvement in 97.2% of 320 pooled patients [2], medication-free relief defined as BNI I or II in 91% of 35 consecutive patients [5], a good outcome defined as BNI I, II, or IIIa in 86.4% of a cohort uniformly BNI V before treatment [8], and pooled relief defined as BNI I or II of 82% across 57 patients, with a wide CI of 50-100% reflecting few contributing studies [6]. Less frequently emphasized is the depth and speed of response: in the one cohort reporting magnitude of change, microsurgical patients improved by three or four BNI points in 64% of cases, whereas radiosurgical patients improved by one or two points in 93% [5], and relief following successful resection is immediate rather than delayed [5,8]. The consistent direction of these figures, despite thresholds that are not interchangeable, is the most reliable feature of the microsurgical literature; their absolute values are not comparable with one another.

Durability is the strongest claim available for resection: the median interval to pain recurrence was 43.75 months following resection against 16.7 months following radiosurgery, a difference reaching significance where the crude recurrence proportions did not [6]. No microsurgical patient experienced aggravation or recurrence over a mean of 50.5 months in one consecutive cohort, in which tumor control was 94% with gross total or near-total resection in 91% [5]. Pooled surgical recurrence was 4.5%, with 10 of 14 recurrences being attributable to tumor remnant or regrowth rather than to failure of decompression [2].

The available evidence suggests, without establishing, that the operative goal is decompression of the trigeminal root rather than completeness of resection as such: release of the root from tumor, adherent vessels, and adhesive arachnoid has been described as sufficient to relieve the neuralgia, and subtotal removal sufficed where mass effect on the nerve was relieved [2]. The principle warrants restraint, since one meta-analysis could not analyze the extent of resection against pain outcome because Simpson grade was inconsistently reported [6], and the predominance of remnant-driven recurrence argues in the opposite direction [2].

Aggregate morbidity figures conceal the distribution that matters clinically. Overall permanent neurological morbidity was 11% in one comparative cohort, but the upper clivus subtype carried 25% permanent abducens palsy together with the lowest rate of complete resection [5]. Pooled surgical complications comprised transient diplopia in 21.1%, facial nerve palsy in 5.3%, and hearing deficit in 3.5% [6]. In a contemporary series of 1000 cerebellopontine angle tumors, not selected for trigeminal neuralgia and reporting no pain outcomes, cerebrospinal fluid leak occurred in 10% and meningitis in 1%, House-Brackmann grade I or II function was retained in 88-90%, and there were no deaths [14]. Postoperative facial hypesthesia is frequent in adherent pathology but characteristically transient, having affected 83 patients across nine studies at rates reaching 67.3% in series dominated by epidermoid cysts, all recovering over several months [2].

Stereotactic radiosurgery and targeting strategies

Tumor control is radiosurgery’s strongest and most consistent property: pooled control was 92.3% across 553 patients with trigeminal schwannoma with no measurable heterogeneity [13], growth control was achieved in all 12 patients of a petroclival series at a median of 5.6 years [4], and control reached 94% in a radiosurgical arm matching its microsurgical comparator [5].

Pain outcomes are considerably more modest and must be reported with their thresholds attached. Pooled complete pain freedom, defined as BNI I, was 38% (95% CI 27-50%), and adequate relief, defined by that analysis as BNI I to III, was 73% (95% CI 63-83%) [3]. An earlier meta-analysis using a different threshold reported initial BNI I in 50.5% and initial BNI I to IIIb in 83.8% for tumor-targeting series; these two measures of adequate relief describe different quantities and are not comparable [7].

Relief is delayed and attenuates. Median latency was four months, ranging from one week to 10 months, in one series [4] and 4.6 months by Kaplan-Meier estimation in another [11]. Initial response rates substantially overstate durable benefit: in pooled tumor-targeting series, initial BNI I of 50.5% was maintained in 36.4% and initial BNI I to IIIb of 83.8% in 41.2% [7], an attenuation also visible within a single cohort followed for a median of 68 months, where adequate relief fell from 83% initially to 50% [4]. Recurrence has been reported at 20-36% across studies [2,5,6,8], and 29% of one cohort underwent subsequent percutaneous balloon compression at a median of four months [11].

The characteristic complication is trigeminal sensory dysfunction, and it is frequently permanent: facial numbness developed in 24% of one cohort and resolved in none [10]; permanent morbidity was 26% in one radiosurgical arm, of which dysesthesia accounted for eight of nine events [5]; and pooled adverse radiation effects were 14% (95% CI 7-22%), with a per-study range from 0% to 43%, indicating that the safety profile in this indication is not established [3]. Pain exacerbation is a modality-specific risk, recorded in 6.6% after tumor-targeted radiosurgery in one synthesis [2] and in 12% of pooled radiosurgical patients vs none after resection [6].

Radiosurgery may be directed at the tumor, the trigeminal nerve, or both; doses cited are those reported by the source studies and are descriptive rather than prescriptive. Figure 1, an original radiosurgical treatment plan from the illustrative institutional experience presented below, shows a session in which both strategies were combined: the isodose line is conformed to the margin of the tumor, while the trigeminal nerve is contoured and treated as a separate target, and the brainstem is delineated as an organ at risk. Tumor targeting, using marginal doses clustering between 12 and 16 Gy typically prescribed to the 50% isodose line [4,5,8], is by far the best-evidenced strategy, accounting for 196 patients in one synthesis [2] and 74 of 81 radiosurgical patients in another [6]. Its weakness is durability, the median change in BNI score at last follow-up being zero [10]; it thus has the most data and the least mechanistic support. Nerve targeting, using 60-90 Gy through a single 4-mm isocenter to the cisternal segment [9,10], is mechanistically more coherent but sparsely evidenced: one systematic review of 517 patients identified a single patient so treated as primary therapy [2]. Results are favorable but preliminary, with nerve-containing treatments having produced the greatest relief at initial follow-up with a median BNI change of -1 [10], and six patients treated after failed tumor-targeted radiosurgery all improving without recurrence [2].

**Figure 1:**
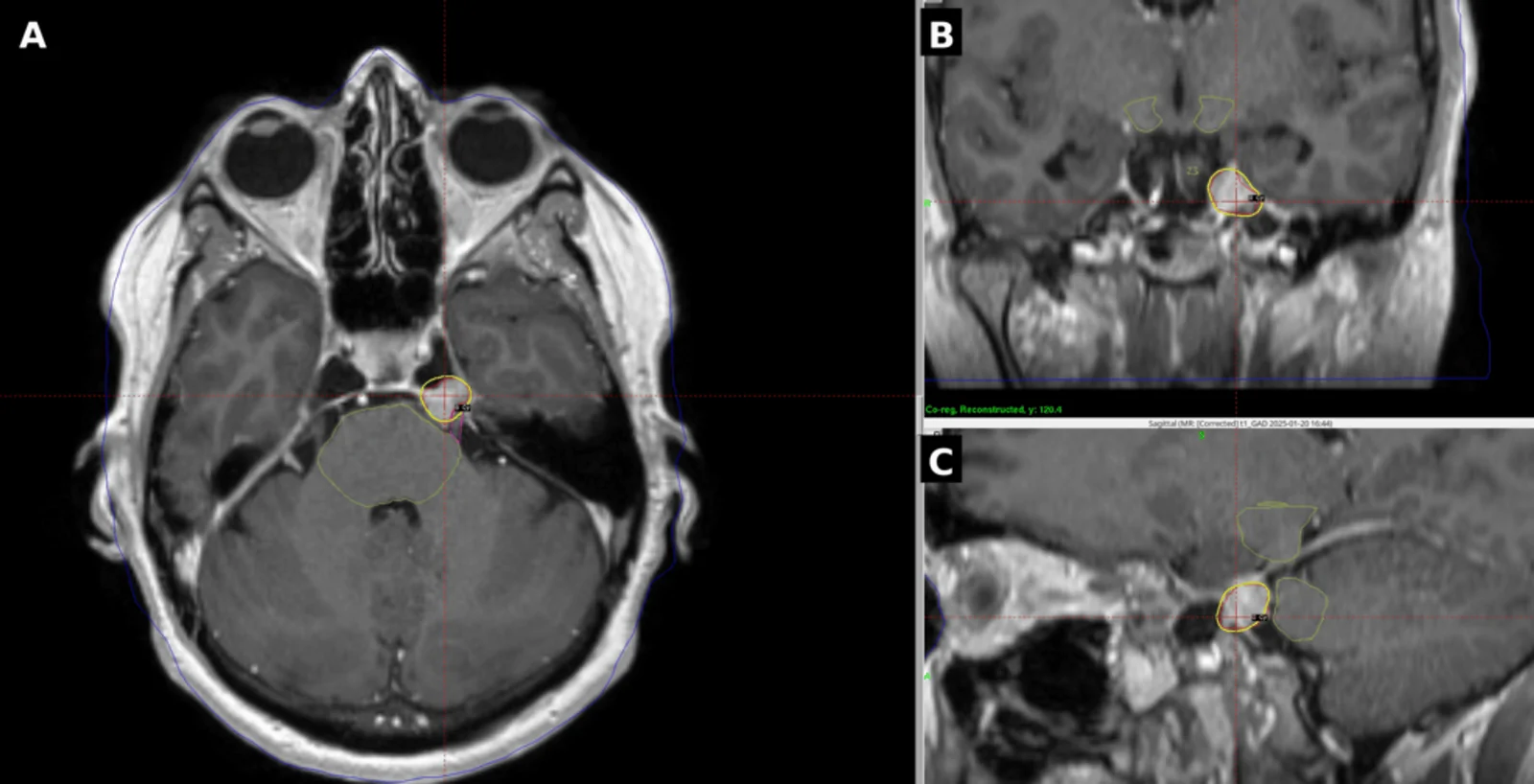
Combined tumor and trigeminal nerve targeting in a patient from the institutional series. Gadolinium-enhanced T1-weighted radiosurgical planning images from patient 4 of the institutional series are presented below, in whom a left cerebellopontine angle meningioma compressed the trigeminal nerve, and both structures were treated within the same session. (A) Axial image showing the tumor contour (red) and the surrounding 8 Gy isodose line (yellow); a separate contour (orange) at the inferolateral margin of the lesion corresponds to the trigeminal nerve target, and the brainstem is delineated as an organ at risk (green). (B) Coronal reconstruction through the same target, with additional organ-at-risk contours shown in green. (C) Sagittal reconstruction through the same target. The blue line in each panel denotes the outer head contour used for planning.

Combined targeting follows from the evidence that the tumor dose does not reliably relieve pain and the nerve dose does not control the tumor, but every reported series is small: six patients in one cohort [10], seven in a pooled analysis [6], and 36 across two to three series in another [7]. On initial relief, no advantage has been demonstrated: pooled initial BNI I to IIIb was 83.7% for dual targeting against 83.8% for tumor targeting [7], and 58% against 29% in a subanalysis that did not reach significance [6]. Durability is the one domain in which a signal emerges, with the median change in BNI score at last follow-up being -2 for dual targeting against -1 for nerve-only and zero for tumor-only; this derives from six patients, all with benign tumors, whereas seven of 10 tumor-only patients had malignant disease, so histology is confounded with target [10]. Safety is contested: complications were reported in 26.7% of dual-targeted patients vs 12.6% with tumor targeting [7], representing four events in a single series of 15 patients. This finding was contradicted by a series that found no excess sensory morbidity [10]. The evidence remains inconclusive.

Where the nerve is radiographically inaccessible, alternative targets have been explored: simultaneous tumor irradiation at 14 Gy with bilateral medial thalamic radiosurgery at a maximum dose of 125 Gy per side has been described in a single patient with complete tumoral encasement and concomitant continuous pain, with relief beginning within 35 hours and sustained over 39 months, the authors noting that a contributory effect from tumor irradiation alone cannot be excluded [12]. This is a single exceptional observation. Taken together, the strategy with the most evidence performs least well on durability, that with the most promising durability signal rests on fewer than 20 patients, and that which is most coherent mechanistically has scarcely been tested as primary therapy; both meta-analyses examining the question have called for randomized comparison of targeting technique [3,7]. How these unresolved alternatives present in practice is illustrated by the institutional experience described below.

Microsurgery vs stereotactic radiosurgery

The studies permitting direct comparison are summarized in Table 4. Observational data associate resection with superior outcomes across several domains: pooled pain relief 82% against 31% (p = 0.004) [6], medication-free relief 91% against 37% (p < 0.01) [5], a good outcome 86.4% against 28% in a baseline-uniform cohort [8], and improvement 97.2% against 79.1% [2]. Responses were deeper as well as more frequent [5], and relief was immediate rather than delayed [4,5,11]. Durability favored resection, with median intervals to recurrence of 43.75 vs 16.7 months [6]. Pain exacerbation occurred in 6.6-12% of radiosurgical patients and in none after successful resection [2,6]. Radiosurgical failure appears salvageable: all 10 patients undergoing resection after failed radiosurgery obtained relief, and surgical reintervention was required in 1.8% of primary surgical vs 19% of primary radiosurgical patients [6]. Taken together, these figures point consistently in one direction, but they are drawn from nonrandomized cohorts using different outcome definitions and should be read as an association, qualified in the paragraphs that follow, rather than as a treatment effect.

**Table 4:** Comparative outcomes of microsurgery and stereotactic radiosurgery in tumor-related trigeminal neuralgia. Primary comparative cohorts report contemporaneous surgical and radiosurgical groups within a single institution; secondary pooled analyses combine surgical and radiosurgical patients drawn from different source studies, so their proportions are pooled cross-study estimates and should not be interpreted as direct comparative effect estimates. Outcome definitions differed substantially between studies and were not harmonized. The four studies are not independent: ref [5] supplies the majority of the surgical and a substantial proportion of the radiosurgical cohort of ref [6], and the surgical arm of ref [8] probably overlaps that of ref [6]. Although pooled pain relief favored microsurgery, the wide CI around the surgical estimate (82%, 95% CI 50-100) indicates considerable imprecision. Follow-up differed between arms in ref [8] (29.7 vs 55.7 months, p < 0.001). S, surgery; SRS, stereotactic radiosurgery

Study	Allocation	n (surgery/SRS)	Pain outcome definition used by the study	Surgery	SRS	p	Recurrence (S/SRS)	Time to recurrence	Tumor control (S/SRS)	Permanent morbidity (S/SRS)
Primary comparative cohorts										
Park et al. (2020) [5]	Patient preference	35/35	BNI I-II, medication-free	91%	37%	<0.01	0%/23%	Not reached/not reported	94%/94% (p = 0.845)	11%/26%
Hegazy et al. (2022) [8]	Convenience sampling	22/25	Good BNI = I/II/IIIa	86.40%	28%	<0.001 (OR 16.3, 95% CI 3.64-72.9)	18.18%/36%	Not reported	100% at 6 months/88% stable	Not separately reported
Secondary pooled analyses (indirect, cross-study)										
Nugroho et al. (2023) [2]	Indirect cross-study pooled comparison	320/196	Improved/unchanged/worse	97.2% improved; 0% worse	79.1% improved; 6.6% worse	Not calculated	4.5%/26.5%	Not reported	Not reported	Not reported
Hallak et al. (2024) [6]	Indirect cross-study pooled comparison	57/81	Pain relief = BNI I-II	82% (95% CI 50-100)	31% (95% CI 18-45)	0.004	6%/20% (p = 0.516)	43.75/16.7 months (p < 0.01)	1/3 progressions (p = 0.06)	Facial numbness 0%/18.5%

Radiosurgery retains clear advantages. Reported tumor control was 94% in both arms of the single cohort, providing a direct comparison [5]; because that is one nonrandomized cohort in which allocation followed patient preference, the similarity is a descriptive observation and does not establish equivalence or noninferiority. The most defensible specific argument concerns anatomy: because upper clivus petroclival meningiomas carried 25% permanent abducens palsy and the lowest resection completeness in the cohort analyzing morbidity by subtype, its authors, while favoring microsurgery overall, suggested that radiosurgery may be preferable for this subtype [5]. Radiosurgery also avoids craniotomy-specific morbidity, with diplopia having occurred in 21.1% of surgical vs 3.7% of radiosurgical patients [6], and remains the consistently recommended alternative for patients unfit for or declining surgery [2,3,6,8].

Two findings qualify the conventional framing. Radiosurgery is customarily presented as the lower-morbidity option, yet in the only included study measuring permanent morbidity comparably in both arms, permanent deficit was more than twice as frequent after radiosurgery (26%) as after microsurgery (11%), the excess consisting almost entirely of dysesthesia of the treated nerve [5]. Radiosurgery avoids the complications of craniotomy; on present evidence, it does not obviously avoid trigeminal injury. Second, the apparent contrast in facial numbness between modalities is substantially an artifact of case mix and reporting: one meta-analysis recorded no postoperative numbness [6], whereas a synthesis containing many epidermoid cases recorded rates reaching 67.3%, all transient [2]. A series of 1000 cerebellopontine angle operations reported no trigeminal outcome at all, illustrating how incompletely this endpoint is captured [14].

These associations cannot be interpreted as causal. No randomized or prospectively allocated comparison exists; treatment was allocated by patient preference in one cohort [5] and by convenience sampling in another [8]. The observed differences between modalities may therefore be influenced by confounding by indication, since patients selected for surgery and for radiosurgery do not represent equivalent populations: they differ systematically in age and fitness, and the studies discussed here further differ in tumor size and location [5,6], histology [2,3], degree of nerve compression and nerve visibility [4], duration of pain and preexisting trigeminal deficit [10,11], prior treatment [6], and center experience [14]. None of the available comparisons adjust for these variables. The magnitude of the association varies from approximately 13-54 percentage points according to the outcome threshold applied and cannot be stated as a single figure. Whether radiosurgery is genuinely the safer modality is not established, with available evidence pointing in both directions depending on whether transient or permanent deficit is considered. Finally, the comparison has not been demonstrated for vestibular schwannoma, epidermoid cyst, or malignant disease.

Treatment selection and special scenarios

Selection is more usefully framed anatomically than by modality preference. The determinants supported by present evidence are operability and comorbidity; petroclival subtype and tumor epicenter, which predict both approach and morbidity [5]; tumor size and mass effect, with open surgery having been favored where neurological deterioration threatens [4,10]; histology; patency of Meckel’s cave and trigeminal nerve visibility, which determine, respectively, whether percutaneous salvage and nerve-directed radiosurgery remain available [4,7,12]; prior treatment; and local availability of skull base and radiosurgical expertise. A published framework recommends microsurgery where gross total resection is achievable and nerve compression is evident, with radiosurgery reserved for patients declining surgery and for deep-seated, high-risk, or inaccessible lesions [3]. No validated selection rule exists, and the framework outlined here should not be read as one.

Small accessible petroclival or petrous apex meningiomas are the best-evidenced situation in which resection is most consistently favored [2,5,6,8], with small upper clivus tumors constituting the one anatomically defined exception on the basis of a single cohort [5]. Vestibular schwannoma-related neuralgia is represented without comparative data, and the radiosurgical signal is discordant: three tumors enlarging after treatment yet accompanied by pain improvement in one series [11], while a constituent study of one synthesis reported worsening in 83.3% of such patients [2]. Trigeminal schwannoma has its own evidence, with pooled tumor control of 92.3% and neuralgia improvement of 63.5% [13]. Recurrent disease is supported by a documented salvage sequence comprising nerve-directed radiosurgery after failed tumor-directed treatment [2], resection after failed radiosurgery [6], percutaneous balloon compression [11], and repeat radiosurgery [9]. Malignant disease, concomitant continuous pain [12], and primary nerve-directed radiosurgery [2] are each represented by fewer than 10 patients, and medically inoperable patients have never been characterized as a defined cohort.

Illustrative institutional experience: individualized targeting in a resource-variable setting

The observations that follow are original data from the authors’ own institution. They are presented separately from the literature discussed above, form no part of that synthesis, and occupy an explicitly lower level of evidence: this is a small, retrospective, single-center case series with incomplete ascertainment, from which no comparative or efficacy inference should be drawn. It is included because it illustrates in practice the targeting problem that the published literature leaves unresolved.

Sixteen patients with tumor-related trigeminal neuralgia were identified by retrospective review of the institutional radiosurgery records covering treatments delivered between 2014 and 2026, a period spanning two successive radiosurgical platforms. Nine, treated during the earlier period between 2014 and 2023, were lost to follow-up, and no outcome data are available for them. Two were treated with tumor targeting alone because their neuralgia was adequately controlled with medication; both were cross-border referrals from Nicaragua for whom no follow-up data were retrievable. The remaining five, treated from January 2023 onward, are those for whom paired baseline and last recorded pain scores were available and are summarized in Table 5.

**Table 5:** Institutional experience with radiosurgery for tumor-related trigeminal neuralgia, January 2023 to 2026. Values are those recorded at baseline and at the last available follow-up. Radiation dose, target volume, follow-up duration, tumor control, imaging response, and medication status were not available for this series and are therefore not reported. Descriptive summary, exploratory only: baseline VAS ranged from 8 to 10, and last reported VAS ranged from 0 to 1, with a reduction in all five patients; the last reported BNI score was I in three patients. These proportions are descriptive and should not be interpreted as efficacy estimates. These five patients are a subset of 16 identified institutionally; nine treated between 2014 and 2023 were lost to follow-up, and two received tumor targeting alone, and none of these 11 are included in the table because paired outcome data were unavailable. Patient 1 is the same patient reported in detail in reference [12]; the two accounts describe one patient and must not be counted as independent observations. BNI, Barrow Neurological Institute pain intensity score; VAS, Visual Analog Scale

Patient	Platform	Target	Trigeminal nerve visible	Targeting strategy	Baseline VAS	Last VAS	Baseline BNI	Last BNI	Adverse effects
1	Infini	Cavernous sinus meningioma encasing the trigeminal nerve; bilateral medial thalamus	No	Tumor + bilateral medial thalamus	10	0	V	I	None reported
2	Gamma Knife Icon	Meningioma; right trigeminal nerve	Yes	Tumor + nerve	10	1	IIIB	IIIB	None reported
3	Gamma Knife Icon	Meningioma; left trigeminal nerve	Yes	Tumor + nerve	8	1	IV	IIIA	None reported
4	Gamma Knife Icon	Meningioma; left trigeminal nerve	Yes	Tumor + nerve	10	0	V	I	None reported
5	Gamma Knife Icon	Meningioma; left trigeminal nerve	Yes	Tumor + nerve	10	0	IV	I	None reported

Reporting of this series follows the conventions appropriate to a small observational case series, and its limitations are stated rather than adjusted for. The institutional experience was reviewed and approved by the Centro Internacional de Cancer, Hospital de Diagnostico Ethics Committee under approval CECIC 2026/002; patient identifiers have been removed, and consent was obtained or waived in accordance with that approval. No patient was excluded on the basis of outcome, and missing data were not imputed: the 11 patients without paired scores are described above but do not appear in Table 5, and this pattern of ascertainment would defeat any attempt to derive a response rate. Adverse effects are reported only as documented in the clinical record at attended follow-up visits; no systematic prospective toxicity grading was applied, so the absence of recorded adverse effects should be read as an absence of documented events rather than as demonstrated safety. Radiation dose, prescription isodose, target volume, duration of follow-up, imaging response, tumor control, and medication status were not consistently recorded in the source documentation and are therefore not reported; their absence is a material limitation of this series and a further reason why it cannot be compared with the published cohorts discussed above.

All five had meningiomas. In four, the tumor and the ipsilateral trigeminal nerve were targeted within the same treatment episode (Figure 1). In the fifth, the patient reported in detail elsewhere [12], a cavernous sinus meningioma completely encased the nerve, which could not be identified on planning imaging; direct nerve targeting was therefore not feasible, and the tumor was treated together with bilateral medial thalamic targets.

Baseline VAS scores ranged from 8 to 10, and last reported scores ranged from 0 to 1, with a reduction in every patient. Last reported BNI scores were I in three patients, IIIA in one, and IIIB in one. There were no adverse effects reported. These are descriptive observations in five patients treated at a single center; they cannot support comparative inference, and no efficacy rate should be derived from them.

One observation nonetheless bears on an argument developed earlier in this review. In the patient whose BNI score remained IIIB, the reported VAS fell from 10 to 1. VAS and BNI capture related but nonidentical dimensions of response, pain intensity, and the composite of pain with medication dependence, so a substantial change in one may coexist with an unchanged category in the other. This discordance, observed in a single patient, illustrates in miniature why the heterogeneity of outcome definitions across the published literature limits comparison between series.

The principal value of this experience lies less in the outcomes than in the targeting problem it illustrates. In four patients, the nerve was identifiable and dual targeting was possible; in the fifth, target selection ceased to be a choice between tumor and nerve and became an anatomical constraint in which the conventional neural target was unavailable. Nerve visibility on planning imaging therefore operates as a practical determinant of which strategies remain accessible, consistent with the published observations discussed above [4,6,12]. Bilateral medial thalamic targeting remains an emerging and highly individualized approach supported by a single published case [12] and should not be regarded as an established treatment for this condition.

The sequencing of treatment was also shaped by context. Where access to specialized radiosurgical care is concentrated in a few centers, and patients travel considerable distances, the feasibility of a staged pathway, such as treating the tumor, observing the pain response over several months, and returning for nerve-directed treatment if neuralgia persists, cannot be assumed. Where anatomy and clinical judgment allowed, simultaneous targeting was therefore adopted as a patient-adapted strategy intended to address the oncological lesion and the pain generator within a single treatment episode. This is a pragmatic rationale rather than a claim of superiority: the published evidence comparing simultaneous with staged or single-target approaches remains inconclusive [3,6,7,10], and the present experience demonstrates feasibility only. The nine patients lost to follow-up illustrate the same constraint from the opposite direction: in a setting where sustained follow-up cannot be assumed, the absence of long-term data is itself a feature of the clinical environment rather than only a limitation of this report.

Evidence gaps and considerations for resource-variable settings

Thirteen of the 14 studies considered are wholly retrospective, one systematic review having identified a single prospective study among the 27 it reviewed [2], and no randomized evidence exists on any question addressed here. The central methodological weakness is inconsistent outcome definition, with at least nine mutually nonconvertible thresholds in use; several apparent disagreements dissolve once thresholds are aligned, and three of the syntheses discussed here call explicitly for standardization [3,7,13]. A related problem is the predominance of proportions reported at arbitrary time points where the decisive quantities are time to relief and time to recurrence; only two of the studies discussed here performed time-to-event analysis [6,11], and these produced the most stable findings available. Further unresolved questions, each acknowledged by its source literature, include the optimal radiosurgical target [3,7], the relationship between extent of resection and pain outcome, unanalyzable owing to inconsistent reporting [6], the contribution of concurrent microvascular decompression [6], and the mechanism of radiosurgical relief [3,7,11]. None of the articles discussed here reports any quality-of-life or patient-reported outcome, and malignant disease and uncommon phenotypes remain insufficiently studied [10,12].

The literature discussed here was generated almost entirely within high-volume specialist centers in high-resource health systems [5,6,10,11,14], and its applicability elsewhere deserves brief consideration; we make no claim regarding regional epidemiology or outcomes, for which no data exist. The relative cost of these treatments is not a fixed property: the only economic analysis within the literature discussed here found radiosurgery approximately three times more expensive than microsurgery in its national setting, its authors explicitly noting that this reverses the pattern reported internationally and attributing the difference to device scarcity and differential financing [8]. Cost ordering is therefore determined by local device economics and health-system financing rather than by the treatments themselves. Radiosurgery’s defining properties also presuppose continuity of care, since relief is delayed [4,11], attenuates over subsequent years [7], and requires salvage in approximately one-fifth to one-third of patients [6,11], a constraint documented in one report in which a staged approach was not pursued partly because follow-up could not be assured after the patient’s return to her country of origin [12]. The same constraint bears on the sequencing of radiosurgical treatment. A staged approach presupposes that a patient can return for a second procedure after an interval of several months; where that cannot be assumed, the choice between staged and simultaneous targeting becomes a question of feasibility and treatment burden as much as of radiobiology. This consideration is absent from the comparative literature, in which staged and simultaneous strategies are compared on outcome alone. Where skull base and radiosurgical expertise is concentrated in few centers, as in Central America, early referral and multidisciplinary assessment assume corresponding importance, and access to high-resolution imaging, which determines whether the nerve can be identified and therefore which treatments are available [4,11,12], cannot be assumed. Treatment selection should remain individualized.

## Conclusions

Tumor control and pain control are separable endpoints in tumor-related trigeminal neuralgia and should be reported and discussed separately; radiographic stability is not evidence of symptomatic success. Observational data associate microsurgical resection with higher, deeper, faster, and more durable pain relief than radiosurgery, with similar reported tumor control, at least in petroclival and petrous apex meningioma; tumor control was compared directly in only one nonrandomized cohort, so this similarity is a descriptive observation and not demonstrated equivalence. No randomized or prospectively allocated comparison exists, allocation in the available cohorts having been determined by patient preference or convenience, and the magnitude of the association varies fourfold according to outcome threshold; the evidence supports a preference, not a proof.

Radiosurgery achieves excellent tumor control with modest pain relief that is delayed and attenuates over subsequent years. Tumor volume reduction does not explain radiosurgical pain relief, and the mechanism remains unknown, which bears on the unresolved question of the optimal target: the strategy with the most data performs least well on durability, and every combined-target series comprises fewer than 20 patients. Treatment selection is best organized around tumor anatomy, histology, nerve visibility, and patency of Meckel’s cave rather than modality preference, though no validated selection rule exists. Evidence-informed management cannot, however, be separated entirely from anatomical feasibility and healthcare context: where the trigeminal nerve cannot be identified, the conventional neural target is simply unavailable, and where a staged pathway cannot reliably be completed, the choice between sequential and simultaneous targeting becomes a question of feasibility as well as of efficacy. A small institutional experience illustrates these constraints but cannot alter the evidence hierarchy. Standardized outcome definitions, time-to-event reporting, prospective allocation, and multicenter data would transform a body of retrospective inference into evidence capable of guiding practice.
